# Financial inclusion for persons with disabilities: Experiences of providers and users of financial products and services in Kenya.

**DOI:** 10.1371/journal.pone.0321493

**Published:** 2025-04-15

**Authors:** Sheru W. Muuo, Bhavisha Virendrakumar, George Okello, Moses Chege, Vibian Angwenyi, Kenneth Gichohi, Hillary Kibet, Sally Nduta, Stevens Bechange, Elena Schmidt, Simon Brown, Emma Jolley

**Affiliations:** 1 Department of Policy and Programme Strategy, Sightsavers, Nairobi, Kenya; 2 Department of Policy and Programme Strategy, Sightsavers, Haywards Health, United Kingdom; 3 Institute for Human Development, Aga Khan University, Nairobi, Kenya; 4 Directorate of Disability Services, Kenyatta University, Nairobi, Kenya; 5 Association of Microfinance Institutions - Kenya, Nairobi, Kenya; 6 United Disabled Persons of Kenya, Nairobi, Kenya; Higher Education Partnership / Erasmus University Rotterdam, ETHIOPIA

## Abstract

**Background:**

Inclusion of persons with disabilities in financial products and services has been identified as an important step on the pathway to achieving the sustainable development goals. Evidence of how people with disabilities are included in financial products and services including strategies to improve their inclusion in low and middle-income countries is limited. We draw on qualitative data to to understand the experiences of providing and using financial products and services from the perspectives of persons with disabilities and representatives of different financial institutions in Nairobi and Migori counties of Kenya.

**Methods:**

Eighty-one persons with disabilities (49.4% <35 years, 57% female) were purposively sampled to take part in 10 focus group discussions. Additionally, 26 in-depth interviews were conducted with financial institution representatives. Data were collected between July and September 2022 and analysed thematically.

**Results:**

Participants described a mixed picture, sharing experiences of different levels of inclusion, and examples of exclusion from different types of financial services and products. Despite good intentions, financial institutions lacked the knowledge and skills required to adapt existing financial products and services, and marketing strategies for different needs and to improve the accessibility of products and services for persons with disabilities. The findings highlighted a need for staff training and establishing institutional policies and guidelines to support the inclusion of persons with disabilities. Persons with disabilities reported embracing digital financial services and constitute a large untapped market for the financial institutions who are able to provide accessible products and services.

**Conclusion:**

Multi-stakeholder approaches are needed to increase disability awareness, develop and enforce policies and guidelines as well as collection of disability-disaggregated data to promote financial inclusion for persons with disabilities in Kenya.

## Introduction

Financial inclusion is defined by the World Bank as individuals and businesses having access to useful and affordable financial products and services (financial transactions, payments, savings, credit, and insurance) that meet their needs delivered in a responsible and sustainable way [1]. Financial inclusion is critical for the achievement of the Sustainable Development Goals (SDGs) [2] since it facilitates access to and use of financial products and services by previously excluded groups such as people in low-income households, microenterprises, youth, women, and persons with disabilities [3]. These include SDG1, on eradicating poverty; SDG 2 on ending hunger, achieving food security and promoting sustainable agriculture; SDG 3 on profiting health and well-being; SDG 5 on achieving gender equality and economic empowerment of women; SDG 8 on promoting economic growth and jobs; SDG 9 on supporting industry, innovation, and infrastructure; and SDG 10 on reducing inequality. Additionally, in SDG 17 on strengthening the means of implementation there is an implicit role for greater financial inclusion through greater savings mobilization for investment and consumption that can spur growth [4].

The Global Findex Database, the world’s most comprehensive database of financial inclusion, estimated that in 2021, worldwide account ownership reached 76% and 71% in developing economies. However, approximately 1.4 billion adults remain unbanked, and it is more common among women, people in rural areas, and those with lower levels of literacy [5]. One group of people that continues being at risk of financial exclusion is persons with disabilities [6].

Persons with disabilities are defined by in the United Nations Convention on the Rights of Persons with Disabilities (CRPD) as those who have long-term physical, mental, intellectual or sensory impairments, which in interaction with various barriers may hinder their full and effective participation in society on an equal basis with others [7]. They account for 16% (1.3 billion people) of the global population and often experience worse outcomes than persons without disabilities in areas such as education, health, and economic opportunities [8].

Persons with disabilities in sub-Saharan Africa have fewer economic opportunities and are more likely to live in poverty due to multiple obstacles in accessing employment [9], finance and public services [10, 11]. Studies in this region reported barriers to financial inclusion such as social stigma and prejudices [12], lack of knowledge of disability among staff working in financial institutions [11], poor digital skills [13], the lack of enabling regulatory frameworks and data on disability needs [14, 15], inaccessible physical infrastructure [16] and communication materials [6]. Despite this, global estimates suggest that persons with disabilities constitute an emerging market of almost 3.4 billion people (including family members and caregivers) with two trillion US dollars in annual disposable incomes [10]. It is further recognised that persons with disabilities are critical to building stronger economies, and that there is a business case for their inclusion in financial markets [10].

In Kenya, the most recent 2019 census revealed that out of the total population of 47,564,296, there were approximately 0.9 million persons with disabilities, translating to 2.2% of the total population [17]. The country has made significant strides in recognizing and addressing the rights and needs of persons with disabilities. In 2003, the Persons with Disabilities Act, the principal legislation on disability in Kenya, was enacted. This Act, with a broadly similar definition of disability as the CRPD, provides for the rights and equalization of opportunities for persons with disabilities and for the establishment of the National Council for Persons with Disabilities [18]. In 2006, the country committed to implementing the CRPD [7]. The current Constitution of Kenya, which came into force in 2010, accords persons with disabilities the right to be treated with dignity and respect, and to access institutions and facilities, public transport and information on an equal basis with others [19].

Over the past decade, Kenya’s financial inclusion has improved dramatically with 79% of adults having bank accounts in 2021, up from 42% in 2011. This leap was largely due to mobile money services [5], which use mobile phones to transfer money, pay bills, withdraw cash, save, process microloans and other services [20]. For example, M‑Pesa, a mobile phone-based money transfer service launched in 2007, spread exceptionally fast and by 2012, had 17 million mobile accounts in Kenya alone. It is argued that through access to microfinance schemes, M-PESA lifted 2% of the Kenyan population out of poverty [21]. However, none of these sources of information include data on disability and thus it remains unclear how these recent advancements in financial inclusion targeting the entire population benefitted persons with disabilities and other vulnerable groups.

Evidence on financial inclusion of persons with disabilities across Sub-Saharan Africa and specifically in Kenya is limited [6]. Studies that are available have focused on either financial service providers (supply-side) [11,22] or users (demand-side) [16,23] and often applied quantitative methods to examine the drivers of exclusion [24–26]. To obtain a more comprehensive picture of financial inclusion it is important to interrogate both the supply and demand sides [27] and to supplement quantitative data with qualitative narratives explored from the perspectives of both financial institutions and persons with disabilities.

For the purpose of this study, we focused on two types of financial products – accounts and loans which are the two most commonly used financial products in Kenya [28]. Financial services were those that related to these two products including account opening, funds deposits, withdrawals and transfers and loan application and repayment. We did not explore other types of financial products available on the market, such as insurance, foreign exchange or investments since these are not commonly used in the low-income areas, where this study took place.

This study aimed to understand the experiences of providing and using financial products and services from the perspectives of persons with disabilities and representatives of different financial institutions in Kenya.

## Materials and methods

### Study design

This study was part of a larger project that focused on financial inclusion of persons with disabilities which involved a rapid review of literature, a qualitative study and a co-creation exercise to develop prototypes of services for financial inclusion of persons with disabilities in Kenya and similar contexts [14]. This paper presents findings of the qualitative study, which included a range of in-depth interviews (IDIs) with representatives of financial institutions and focus group discussions (FGDs) with a sample of persons with disabilities.

### Study setting

The study was conducted in two sites, in Kenya’s capital Nairobi, an urban site, and Migori county, a rural site. According to the 2019 national census the number of persons with disabilities was higher in rural areas with 2.6% or 0.7 million people in rural areas and 1.4% or 0.2 million people in urban areas having a disability [29].

Nairobi is the largest city and as a financial and commercial hub, it hosts the highest concentration of financial institutions and industries in the country [30]. Migori is situated in South-Western Kenya; the main economic activities in the county are agriculture, fishing, manufacturing and mining. The county faces numerous challenges including high poverty levels, high unemployment rates, droughts and poor infrastructure [31]. Out of the 916,692 persons with disabilities in Kenya’s 47 Counties, the proportion that resided in Nairobi was 4.6% while those who were from Migori were 3.2% [29]. Migori was chosen as the rural area in the study based on convenience because Sightsavers, the iNGO that led this work, has presence there.

### Study population

Focus group discussions were conducted with persons with different impairments, aged between 18 and 60 years, recruited by purposive sampling [32]. The IDIs targeted financial institution representatives, who worked as senior managers in various capacities including branch, general or business development managers who were knowledgeable about financial products and services offered by their institutions. These financial institution representatives represented 16 microfinance institutions (MFIs) and one bank, all of which were members of the Association for Microfinance Institutions of Kenya.

Out of the 16 MFIs, there were nine deposit-taking microfinance institutions (or microfinance banks) and seven non-deposit or credit-only microfinance institutions. Defined by the 2006 Microfinance Act, microfinance banks are companies that are licensed by the Central Bank of Kenya to carry out microfinance banking services including branches, outlets and any other licensed places of business. They accept deposits, register accounts, accept cheques, provide loans and carry out investments on behalf of their clients. Credit-only microfinance institutions are companies that provide lending services but do not take cash deposits or collateral from their clients [33].

### Sampling

One hundred and seven participants including 81 FGD and 26 IDI participants took part in the study. Ten FGDs (six in Nairobi and four in Migori) were conducted with 81 persons with disabilities recruited with support from the United Disabled Persons of Kenya (UDPK), the umbrella body for Organisations for Persons with Disabilities (OPDs) in Kenya. The UDPK mobilised study participants through their member OPDs in Nairobi and Migori. Each FGD had six to nine participants.

Twenty-six representatives (17 in Nairobi and 9 in Migori) from 17 financial institutions were also selected purposively with support from the Association for Microfinance Institutions of Kenya. Microfinance Institutions were primarily targeted for this study, as they work more with low-income households and micro, small and medium enterprises (MSMEs) than banks, particularly in rural and peri-urban areas [34].

### Data collection

Data were collected between 27^th^ July and 23^rd^ September 2022. The FGDs and IDIs were guided by the topic guides developed based on the rapid literature review [14]. Focus groups were used in place of individual interviews for persons with disabilities to allow participants to feel empowered and share their experiences among other persons with disabilities. Two researchers with qualitative research experience conducted IDIs and FGDs in either English or Swahili depending on participants’ preferences.

The IDI and FGD guides had questions on financial products and services available to clients in general and specifically to persons with disabilities, institutional policies and guidelines, marketing of products and services, accessibility of products and services, staff knowledge and attitudes as well as awareness of disability and client’s additional needs. All IDIs and FGDs were audio-recorded.

### Data management and analysis

All IDIs and FGDs were transcribed verbatim, and participants identifiers anonymized during this process. Data were analysed thematically and managed using NVivo 12 software [35] in line with the procedures for analysing qualitative data proposed by Braun and Clarke [36]. At the first level of analysis, transcripts were read thoroughly by SM and VA, who developed and agreed on the code book. Using the code book, the remaining data were coded by VA, in consultation with SM and BV. Following the coding, VA with support from SM and BV organised the codes alongside broader themes. A general inductive approach for analysis of qualitative evaluation data was used [37].

A validation workshop was held in October 2022 with representatives of both participant groups to share and validate the study findings, brainstorm and identify key barriers that needed to be prioritised and identify potential solutions and disability-inclusive recommendations for further deliberations during a programmatic co-creation workshop [14].

### Ethical considerations

Ethics approvals were obtained from the Kenyatta University Centre for Research Ethics and Safety (PKU/2454/E1585) and the National Commission for Science, Technology and Innovation (NACOSTI/P22/17185). Written informed consent was obtained from all participants prior to data collection. The consent form was read and explained to participants while responding to questions when they arose. After orally consenting to participation, participants signed and dated the form.

Special considerations and accommodations were made to facilitate participation of persons with disabilities. For example, the FGDs were held in buildings which were convenient and accessible for participants with mobility limitations. Persons with visual impairments brought along their carers or guides. Sign language interpreters were available for persons with hearing impairments to ensure accessibility of information.

### Inclusivity in global research

Additional information regarding the ethical, cultural, and scientific considerations specific to inclusivity in global research is included in the Supporting Information (S1 Checklist)”

## Results

### Participant characteristics

One hundred and seven participants, including 81 persons with disabilities and 26 representatives of financial institutions participated in this study. Among persons with disabilities, half (49.4%) were young people aged 18–35 years, and the other half were aged 36–60 years. There were slightly more women than men at 56.8% and 43.2% respectively. More participants were from Nairobi (59.3%) than Migori (40.7%). The largest proportion of participants had physical disabilities (39.5%) followed by those with visual impairments (13.6%), hearing, speech and language disabilities (12.3%) as well as albinism (4.9%). Approximately 3.7% had mental health disorders, intellectual disabilities and autism spectrum disorders while 2.5% had multiple disabilities. Some participants (23.5%) preferred not to disclose their disability status on paper despite discussions around confidentiality before data collection. Disability categorization was based on the Kenyan 2022 Disability Medical Assessment and Categorization Guidelines [38].

Among persons with disabilities participating in the study, over half had an account with a formal financial institution. One in five (21%) had experience of taking a loan from a financial institution, while about a third (32.1%) had borrowed money via a digital lending platform.

In the IDIs with financial institutions representatives, there were more male (69.2%) than female (30.8%) participants. Half of them had work experience of 11 years and above (50.0%) and almost half (46.2%) had been working for their current employer for five years or less.

### Experiences of providing and using financial services

The themes rising from the analysed have been grouped under six broad categories presented below.

#### Financial products and services available in the market.

Financial institutions representatives described various products and services available to their clients. These included individual, joint and group bank accounts, a range of loan and credit facilities, such as local purchase order (LPO) financing, asset financing, agricultural loans, school fee loans, and small and medium enterprise (SME) investment loans. The majority of representatives from financial institutions reported that their products and services targeted the population as a whole, although they did have products for specific ages (e.g., young people account) or professional groups (e.g., construction financing, agricultural loans). Only one institution aimed at financially empowering persons with disabilities through specific products designed for these clients, as described by their representative:

*“Our core business is lending, and our area of specialization is LPO [Local Purchase Order] financing. Our main target customers are youth, women and persons living with disabilities. So, they make the bigger part of our clients in terms of financial services. We lend them, if they are able to do their work, then they pay us back, once they are awarded an LPO or a tender.”* (Credit only microfinance, urban)

Participants with disabilities appeared to be more knowledgeable of banks than MFIs and only a few knew the difference between them. The most common financial products used by these research participants were bank accounts, loan and credit services, particularly asset financing and school fee loans.

Some persons with disabilities had no accounts. The main reason for this was low or irregular income. These participants preferred to store the little cash they had in a safe place in their house, as one participant explained:

*“I repair electronics such as radios, TVs, computers… Unfortunately, the income is very little. It’s what we call from hand to mouth sort of work. Therefore, it’s very unlikely for me to put money in a bank.”* (Younger participant with account, rural)

Many participants with disabilities showed high levels of awareness of digital platforms for saving and borrowing money. The services most frequently named were M-Pesa (a mobile phone-based system that enables users to deposit, send and withdraw funds), M-Shwari (a savings and loan service), and Fuliza (an overdraft facility) provided by Safaricom, the leading Kenyan telecommunications company. Other digital lenders mentioned included Silicone Valley backed Tala and Branch as well as Zenka, owned by the Norwegian software maker, Opera. A number of participants preferred using these digital services instead of bank or MFI accounts, as one research participant explained:

*“I can say that I prefer M-Pesa because I can save my money there and get a loan from there. I can also use it as a fixed account.”* (Younger participant with no account, urban)

Approximately third (30%) of the persons with disabilities interviewed were members of Savings and Credit Cooperative Societies (SACCOs) and/or *Chamas*, where members of a community group come together to save and invest for the benefit of all members. For some participants, these schemes were more popular than formal financial services due to their less cumbersome registration processes, lower interest rates, faster loan processing times, flexibility in repayment periods, and the presence of co-guarantee systems where fellow members served as loan guarantors, as one participant pointed out:

*“…the interest rate, you will find that it is higher in the banks and lower in the Chama, so some… prefer the Chamas to the banks.*” (Older participant with no account, urban)

### Institutional policies and guidelines

Most financial institutions interviewed reported having no specific policies or guidelines on disability including the MFI that has a focus on persons with disability as reported below.

“*No, I don’t think we have any documented policy. What is noted in our policy is that we focus on that group [persons with disabilities] and then I think it leaves it at that. I know that in the coming days that is where we intend to go back to. We probably need to formulate our policies to address their needs more.”* (Credit only microfinance, urban).

Research participants reported they relied on broader customer service policies and human resource policies on non-discrimination of clients and staff. Only one financial institution mentioned having an operational policy on loan procedures which detailed terms and conditions for working with persons with disabilities. Another financial institution described how they had to adjust their terms of service when one of their clients developed severe physical limitations due to a terminal illness.

A few research participants reported having a disciplinary system to handle any reported cases of discrimination or poor handling of clients. This system included provisions on discrimination of clients on the ground of disability.

### Marketing of services and products

Many institutions also pointed out that their marketing and communication strategies targeted the general population, and they did not know whether the printed materials, websites and social media platforms they used were accessible to persons with disabilities:

*“...you see as a marketing guy, our business is…visibility and branding outside there.... we are expected that anytime we post in print, it also goes on social media...the question that we need to ask ourselves is…out of the guys that are blind and those …having issues physically, do they have that opportunity of reading it? Can they access these newspapers? Do they have mobile phones?*” (Microfinance bank, urban)

Several institutions, however, said that they had made specific efforts to reach clients with disabilities. The most common approach was word of mouth to recruit new clients with disabilities. This involved persons with disabilities, who were already product users sharing information with other persons with disabilities that they personally knew. Another approach was to send field agents to the homes and businesses of clients with disabilities. This was particularly common in promoting new financial products and loans. A few institutions reported that where necessary, these agents were accompanied by sign language interpreters:

“*Our way of marketing is very personal…we meet with them [persons with disabilities] and talk to them… if there is a need for sign language, I bring in a sign language interpreter.”* (Credit only microfinance, urban)

Persons with disabilities reported they needed more information about different products and services available; they wanted more information and training on how to access them and how to use them safely and beneficially. They recommended financial institutions work with persons with disabilities in the design of such products and services and training sessions.

### Accessibility of products and services

Several persons with disabilities reported that the long distances and travel time to get to a financial institution was a major challenge for them. Those who needed aides had to cover costs of travel for themselves and for people accompanying them, which many could not afford:

*“For you to have access to the money, you have to tag along your caregiver and maybe the bank is not near; so maybe you have to look for [bus] fare...and sometimes you have to get enough money for a cab to and from [the bank]. And you see, many are not employed and maybe depend on well-wishers and family.”* (Younger participant with account, rural)

Representatives of financial institutions also described their efforts to make their products and services more accessible to people with additional needs. They specifically mentioned voice-enabled mobile banking applications and screen readers for website content. When persons with visual impairments visited their institutions, staff read documents out loud. For persons with intellectual challenges, discussions were recorded and shared in an audio file. Persons with hearing impairments were provided with information using handwritten notes and text messages; they could also bring in sign language interpreters for communication support.

When describing their physical premises, some financial institution representatives said that they tried to make structural adjustments to improve access for persons with disabilities. These included locating offices on the ground floor, installing ramps, having wide corridors, installing lifts with voice-enabled features and Braille imprinted keys, lowering the counters for wheelchair users and installing accessible toilets:

*“...we … made our ATMs accessible, banking halls accessible, we have also made sure that there are counters that are sunken to a level that you can …work comfortably from your wheelchair … we have 19 branches, they are all friendly to persons with disabilities.*” (Microfinance bank, urban)

However, several financial institution representatives acknowledged that their buildings were inaccessible for persons with mobility difficulties. They explained that many offices were rented spaces, which presented challenges with making structural adjustments. Any changes to office layouts required negotiations with landlords and certain structural modifications required special authorisations. This problem was more common among institutions in rural areas, which had limited choices of buildings for rent:

*“We prefer most of our branches to be on the ground floor but [in] some places, it is difficult to get good spaces.”* (Credit only microfinance, urban)

Some financial institutions argued that the cost of making their premises and products more accessible was a major challenge for them. They specifically mentioned high costs of printing existing forms using large fonts, preparing documents in Braille, hiring sign language interpreters, modifying mobile applications and making accessibility adjustments to the buildings.

Opinions of participants with disabilities on accessibility of financial institutions and their products varied. Many said that inaccessible infrastructure was a major barrier preventing them from more frequent use of financial institutions. They specifically mentioned glass dividers, which separated clients and banking staff and created difficulties in communication for persons with hearing impairments. Many buildings lacked lifts and ramps, and the floors were often slippery. Sometimes counters and Automated Teller Machines (ATMs) were too high for persons with physical impairments or those of short stature.

The lack of accessible information was also mentioned as a major challenge. Participants with disabilities said that most institutions did not have sign language interpreters and had to exchange information using written notes, which was strenuous for both the clients and the staff. Some said that the staff often could not understand the written notes due to the differences in grammar used by people, who communicated in sign language:

*“When you get there, the forms that are supposed to be filled, the English [language] used is not simple and direct because of our grammar structure… sometimes when we write to them, they think we are stupid because we are using incorrect English but that is what our grammar is based on.”* (Younger participant with account, urban)

Participants with visual impairments also said that the documents they had to complete used small font size and there were no forms in Braille for those who were blind. Many had to rely on their guides in reading the documents. Those who had low literacy levels or had intellectual challenges found that the language used in many documents was too technical and difficult to understand.

While digital financial services were popular among many participants with disabilities, some experienced difficulties with installation and update of mobile applications due to their inaccessible formats. Major difficulties were experienced by persons with visual impairments with the applications, which did not have audio-enabled features:

*“...if we say that somebody is visually impaired then yes, we have a USSD code but they can’t dial it because they can’t see.”* (Credit only microfinance, urban)

Financial institution representatives acknowledged the need for better training of their staff on how to develop and improve digital channels for persons with disabilities.

Another common concern expressed during the interviews with both persons with disabilities and staff of financial institutions was the security of digital channels, data privacy and third-party access to sensitive information. Persons with visual impairments were concerned that their phones could be used by someone to process loans without their consent. To prevent unauthorized use of mobile services persons with visual impairments had to provide details of their designated aides who would support them in conducting financial transactions which introduced another layer of complexity and prolonged the account registration process. Some participants, who could not use ATM services due to their high position also were concerned about security of their transactions, as they had to share sensitive information with their aides:

*“…for some of us to access the ATM…you have to rely on someone else to enter for you the PIN and it is sensitive, sharing a PIN…So...if it [ATM] was placed on a level that I can do my transactions then I wouldn’t need help with PIN.”* (Younger participant with no account, urban)

Financial institution representatives corroborated these concerns. They acknowledged that persons with disabilities were at high risk of fraud, as one interviewee explained:

*“I think what they [persons with disabilities] face is the intermediaries that come with them, who would want to take advantage of them. They probably want to get a little something for themselves and they [persons with disabilities] have already trusted them out there, so they come, access funds and after a while they [persons with disabilities] tell you that that the guy [aide] took their money and went away …. So … they [persons with disabilities] are not paid and next time you are not going to lend them because you still have not recovered your money.”* (Credit only microfinance, urban)

Some persons with disabilities mentioned the presence of ramps, lower counter tops, designated rooms or desks for clients with disabilities, fast tracking in queues and ATMs and mobile applications with audio-enabled features. But all agreed that more needed to be done:

*“…most of the banks nowadays are trying to be considerate of persons with disabilities and especially when it comes to ramps …The deaf and someone …. visually impaired …[can] ask someone to help them.”* (Younger participant with account, rural)

## Staff knowledge and attitudes

Most financial institutions said that their staff was helpful and accommodating to clients with disabilities and their needs. They specifically mentioned security guards, who guided clients with disabilities through their premises. They also said that clients with disabilities were prioritised in queues and staff helped with filling in forms and operating ATMs. Some institutions mentioned that their staff serving at the customer desks were trained in attending to clients with additional needs.

Some interviewees however admitted the existence of stereotyping attitudes where persons with disabilities were perceived to be a high risk, as a borrower or a guarantor, as one interviewee recalled:

*“I had a woman [client] who wanted a loan, but the husband was disabled, and he was supposed to be the guarantor. So, questions on whether the guarantor can repay the loan in case she defaults arose… it is not easy to give them [persons with disabilities] loans as compared to others because you wonder if they can conduct … business.”* (Microfinance bank, rural)

Participants with disabilities also pointed out to a widely spread perception that persons with disabilities did not have money to regularly use financial services. Some reported that they often felt ignored or dismissed while others reported being labelled as “sick people” or “thieves”. This is how one research participant recalled their experience in a bank:

*“What made me not to open an account is…I went to [this] institution … several years ago…The first thing they asked me was where I would get the money to keep in the account. Then they started attending to people who didn’t have disability. I waited for a while but then got tired of waiting, so I left.”* (Older person with disability, rural)

Some participants argued that one of the reasons why they preferred to use mobile money services is that they wanted to avoid physical contact with financial institutions, so that their disability remained secret which then prevented potential stigma and discrimination.

## Awareness of disability and clients’ additional needs

Almost all financial institutions interviewed reported limited awareness of disability or how to support persons with disability related needs. Most staff did not know how to communicate with persons with disabilities or how to ensure that marketing materials and products met accessibility requirements. Some interviewees were concerned that their focus on clients’ additional needs may be interpreted as patronizing and discriminating. For example, one interviewee recalled a situation when they wanted a client with disability to be served ahead of the queue. The client felt offended and angry, as they did not want to be treated differently from others.

Most financial institution representatives said that they had not received any training on disability. The only training they mentioned was sessions on diversity of clients, which included persons with disabilities, as part of their general on-the-job training. There were, however, two exceptions. One financial institution organised regular fortnight meetings with staff across all their branches to discuss equity and inclusion, which included topics on disability. Another institution conducted two training sessions for their staff on basic sign language, disability awareness and etiquette. The training was done in partnership with a local organization of persons with disabilities (OPD):

*“...we have had two training [sessions] where the staff learnt some basic alphabet and communication in sign language. I think now …we will be more exposed…and maybe the management will see the need to incorporate those trainings….”* (Credit only microfinance, rural)

Additionally, the majority of financial institutions interviewed were unaware of the proportion of their clients that had disabilities or accessibility needs, as one participant explained:

*“We are not collecting data on who is disabled or not. So, I’m not even sure how many persons with disability we have and what disabilities they have.”* (Microfinance bank, urban)

Participants explained that they mainly discovered their clients’ disabilities when they interacted with them and when difficulties in performing certain activities were either evident through interactions or reported by the client. Only a few institutions said that they tried to systematically record information on disability, when the clients either presented their disability card or their tax exemption certificate during account registration processes. Only a handful of participants interviewed were aware of these documents and proactively asked whether their clients had them.

Financial institution representatives raised concerns about potential risks arising from inappropriate collection or storage of disability data. Some argued that if the disability status is known to the institution, clients may be stigmatised or discriminated against:

*“...we don’t categorise clients like that but if someone has eye problems, some have leg problems and they are involved in business, they get support from us...You know when you collect that [information], discrimination may occur, so we don’t [do] that... We don’t segregate them [persons with disabilities] and that is why we don’t keep such information.”* (Credit only microfinance, urban)

Opinions of participants with disabilities on recording their disability status by financial institutions varied. Some thought that this information could ensure better accessibility and more support from the staff. They also argued that this could potentially support the development of products and services specifically for persons with disabilities, for example loans with lower or waived interest rates. Research participants from financial institutions were hesitant about the feasibility of such products, as they could be considered discriminatory. Also, eligibility for such services would be difficult to establish without a formal verification process (e.g., disability cards), which could potentially open avenues for abuse and fraud.

*“I don’t think it’s necessary because one might pretend to have a disability… so they be given a loan.”* (Younger participant with no account, urban).

## Discussion

This is one of a few studies which explored the experiences of inclusion of persons with disabilities in financial markets in Kenya. Our data shows that Kenya has made significant progress in increasing access to financial services for the general population and made efforts to facilitate access for vulnerable groups, including persons with disabilities. Among our participants with disabilities, half had access to a financial account, one in five borrowed money from a formal financial institution and one third took loans from a mobile lender. Although not nationally representative, the study showed that many persons with disabilities could access financial products and services available in the market.

Our findings are consistent with the recent data from the World Bank, who reported that Kenya was at the forefront of financial inclusion in sub-Saharan Africa region with 79% of the general population reporting access to financial institution or mobile accounts. However it is important to note that although the World Bank recognises socio-economic inequalities in access to financial services and disaggregates its global data by six individual characteristics (sex, age, residency, education, workforce and income), disaggregation by disability status is lacking and financial inclusion of people with disabilities continues to be a significant knowledge gap [5]. Data from our study suggests that persons with disabilities may be more disadvantaged than their non-disabled counterparts. However, as it was a qualitative study, we cannot quantify the actual access gap. Moreover, the World Bank global database [5] and data specific for Kenya [25] suggest that women, people with lower levels of education, those out of work and those with lower income are less likely to have access to financial services. As all these characteristics are also associated with disability [8], it is possible that people with disabilities experience double or triple disadvantage due to intersection of disability with other vulnerabilities.

Financial institutions that participated in our study also described their efforts to make their infrastructure and products more accessible for people with disabilities, although there were variations between institutions and research participants. Our study identified a number of barriers faced by financial institutions in improving access to their services by persons with disabilities. These include poor knowledge of disability and clients’ additional needs among staff, the lack of information about disability status of their clients and high costs of structural adjustments to their premises. Similar results were found in other studies in Ghana [15], Uganda [23] and Bangladesh [39, 40].

Literature also describes examples of significant improvements in financial inclusion as a result of implementing more disability inclusive practices. For example, a study in Uganda demonstrated that implementing interventions targeting discriminatory attitudes and behaviours towards persons with disabilities among staff of microfinance institutions significantly increased the number of their clients [41]. These interventions were developed through involving persons with disabilities in the in the product design, marketing approaches and more accessible infrastructure. The interventions showed also their effectiveness in building confidence and increasing self-esteem among persons with disabilities involved [41].

Banks in Kenya have also made progress towards disability inclusion guided by the efforts of the Kenya Bankers’ Association (KBA) which adopted financial inclusion for persons with disabilities as a strategic goal in 2019. Since then, KBA with support from partners developed a roadmap for the banking industry to training for staff on how to support persons with disabilities, recruit more staff with disabilities, improve accessibility in their branches, and improving accessibility of mobile applications, online banking, and printed and electronic documents [11]. However, it was clear from our findings that these strategies had not yet been rolled out by microfinance institutions, who were the primary participants in our research.

Available literature is also clear that financial institutions need to work closely with organisations of persons with disabilities to develop and implement guidelines on how to support persons with disabilities (17). These organisations can help financial institutions develop effective and sensitive approaches to marketing, accessible infrastructure, and collecting disability data. It would also be beneficial for financial institutions to work with disability advocacy groups and policymakers to ensure financial products and services are designed with the unique needs of persons with disabilities in mind, thereby promoting greater their economic independence and participation in the financial system for this underserved populations [14]. These partnerships are essential for truly inclusive financial markets based on the principle “Nothing about us without us” and will benefit both financial institutions and their clients [42].

Our research findings highlighted that both financial institutions and persons with disabilities had embraced digital technology. This was encouraging especially since inclusive digital technology has been shown to be an important pathway to financial inclusion for persons with disabilities (7). Evidence from previous studies also showed that the ownership of mobile phones [13] and accessible digital products facilitated access to financial services by clients with additional needs [43]. Financial institutions therefore should work closely with mobile network operators, fintech providers and persons with disabilities in designing digital financial products, their delivery mechanisms and communication strategies [44]. This should be accompanied by training on data security and safety to empower persons with disabilities and prevent them from becoming victims of fraud or other financial crimes.

This study highlighted the importance of understanding the accessibility needs of clients which requires collection of meaningful disability data at the institutional level. Disaggregation by disability status of financial data collected through global, regional and national surveys should also be improved. This is important for identifying population groups vulnerable to exclusion and for monitoring and evaluating progress towards more equitable financial access. This more granular understanding of the populations at risk of financial exclusion will allow for better planning and budgeting of reasonable accommodation. If persons with disabilities remain invisible in data, they remain unaccounted for [45].

In Kenya, financial inclusion monitoring is supervised by the Central Bank of Kenya (CBK) [46]. The Central Bank can guide financial institutions in the collecting disability-related data to be able to fulfil its commitment to the Inclusive Data Charter signed in 2018 which included the country’s commitment to promote collection of accurate data on persons with disabilities for use in planning and programming and to ensure no one is left behind [47]. Central Bank can also guide financial institutions in the development of institutional level policies and guidelines on disability inclusive services and to facilitate their enforcement.

We acknowledge several limitations in our study. First, the study was vulnerable to social desirability bias. Even though financial institution representatives were assured of data confidentiality, it is possible that some research participants provided a more positive picture in the attempt not to discredit their financial institution. This may have been affected by the fact that most of these interviews took place on the premises of these institutions. Second, the study did not include larger and more popular commercial banks or the Central Bank of Kenya despite multiple attempts to involve them, largely due to their lengthy institutional approval processes. We are aware from the literature that some of these institutions have made significant strides towards disability inclusion, but their perspective could not be fully reflected in our research (12). Thirdly, the study involved persons with disabilities from low-income areas in the two counties which was not representative of all persons with disabilities within these counties or in the country. We recommend further research involving persons with disabilities from different settings in Kenya to get a more wholistic picture. Additional quantitative research is also recommended in order to draw further conclusions indicating differences in financial inclusion among people with different types of disabilities, between women and men and other sub-categories. Fourthly, mobilisation of participants with disabilities was done through the national umbrella organisation for persons with disabilities. It is possible that our participants were more knowledgeable about disability and financial services and their views may not have been representative of all people with disability in Kenya.

In conclusion, our study showed that although a significant progress towards financial inclusion has been made in Kenya in recent years, more needs to be done to ensure access by vulnerable groups, including persons with disabilities. It is envisaged that these insights will inform financial institutions on how to adapt existing financial products and services to better serve persons with disabilities and ensure financial inclusion for all.

## Supporting information

S1 Checklist Checklist Inclusivity in global research. (DOCX)
